# Investigation of Virulence Genes of *Staphylococcus aureus* Isolated from Sterile Body Fluid Samples and Their Correlation with Clinical Symptoms and Outcomes

**DOI:** 10.1155/2021/5354747

**Published:** 2021-12-26

**Authors:** Tao Lin, Qianhui Li, Defu Jin, Wanbo Liu, Chaogui Tang, Xiaoyun Zhang

**Affiliations:** Department of Medical Laboratory, The Affiliated Huaian No. 1 People's Hospital of Nanjing Medical University, Huanghe West Road 1, Huai'an, Jiangsu 223300, China

## Abstract

*Staphylococcus aureus* is the major pathogen causing nosocomial human infections and produces a variety of virulence factors that contribute to its ability to colonize and cause diseases. This study was conducted to investigate the virulence genes in *S. aureus* isolated from sterile body fluid samples and their correlation with clinical symptoms and outcomes. The VITEK 2® Compact system was used to perform biochemical identification and antimicrobial susceptibility tests on 33 *S. aureus* isolates. Virulence genes were amplified using multiplex PCR. The virulence gene patterns were analyzed by systematic cluster analysis. The frequency of methicillin-resistant *S. aureus* was 45.45%, and 17 virulence genes were identified. Genes encoding hemolysins showed high frequencies. The frequencies of *hla*, *hlb*, *hld*, and *hlgB* were 93.94% and that of the *luk-F/S-PV* was 21.21%. Except for the frequency of *splB* (51.52%), the remaining genes encoding invasive proteases showed frequencies greater than 81.82%. Among the patients, 100.00% had undergone invasive medical procedures and 24.00% had been treated with more than three types of antibiotic drugs. Invasive medical procedures are the main causes of infection. Resistance to antibiotic drugs and the status of carrying virulence genes were highly related to clinical symptoms and outcomes.

## 1. Introduction

*Staphylococcus aureus* is a common opportunistic pathogen that causes large numbers of infections because of the presence of its virulence factors and high resistance to most antibacterial drugs [1]. It is the leading cause of lethality in hospital- and community-acquired infections [2]. The relationship between *S. aureus* colonization and pathogenicity and human diseases has been extensively investigated [3, 4]. Approximately 500,000 *S. aureus*-related infections occur annually in the United States [5]. *Staphylococcus aureus* is also one of the most common and fatal causes of blood infection, the incidence of which is increasing [6].

Different *S. aureus* strains carry different virulence factors and cause varying pathogenic characteristics, resulting in different diseases [7–11]. Recent clinical research has focused on the relationship between virulence factors, bacterial evolution, and host factors [12, 13]. Virulence factors in *S. aureus* include hemolysin, leukocidin, invasive proteases, hyaluronidase, lipase, nuclease, and staphylokinase. Hemolysin plays an important role in colonization and pathogenicity [14, 15] by creating holes in the host cell membrane, enabling water and other toxic factors to flow into the host cell and cause cell swelling, rupture, and necrosis [16, 17]. Some domestic scholars showed that hemolysin also has proinflammatory and apoptotic effects [13]. Foreign research revealed that hemolysin can also cause severe pneumonia. The genes encoding hemolysin include *hla*, *hlb*, *hld*, *hlgA*, *hlgB*, and *hlgC*. Leukocidin exerts destructive effects on host blood cells (including leukocytes), skin, and mucosal cells, leading to inflammatory reactions [18]. The genes encoding leukocidin include *luk-F/S-PV*, *lukE*, *lukM*, *psm-mec*, and *psm-α*. Invasive toxins include invasive proteases, staphylokinase, hyaluronidase, lipases, and nucleases, which degrade a variety of macromolecules in host tissue cells, allowing inflammation to spread to deeper parts of the body. Staphylokinase, also known as plasmin, induces the spread of fibrinolytic bacteria. Hyaluronidase can lyse the extracellular matrix to induce inflammation spreading. Lipase expression products can degrade fats and oils, which are beneficial for the colonization of bacterial strains [19]. Nucleases produced by *S. aureus* can hydrolyze nucleic acids (including DNA and RNA) in host cells. The genes encoding invasive toxins include *sspA*, *splB*, *splC*, *splB*, *sak*, *hysA*, *lip*, and *nuc*.

We have been continuously monitoring the drug resistance genes and virulence genes of *S. aureus* isolated from clinical samples at the Affiliated Huaian No.1 People's Hospital of Nanjing Medical University since 2013 [20–22]. In this study, we investigated the virulence genes of 33 *S. aureus* strains isolated from sterile body fluid samples of patients in our hospital and investigated the relationships between methicillin-resistant *S. aureus* (MRSA), invasiveness, clinical symptoms, and outcomes.

## 2. Materials and Methods

### 2.1. Bacterial Identification and Antibiotic Susceptibility

All 33 *S. aureus* strains were isolated from sterile body fluid specimens of inpatients at the Affiliated Huaian No. 1 People's Hospital of Nanjing Medical University from April 2015 to November 2019 and stored at −80 °C. Blood samples accounted for 84.85% (28/33) of the total samples, and pleural effusion samples accounted for 18.18% (5/33) of the total samples. The strains were isolated, cultured, and identified in strict accordance with the “National Clinical Laboratory Operating Procedures.” All isolates were initially identified based on the colony morphology, Gram staining, and coagulase tests performed at the clinical microbiology laboratory. The results were confirmed using an automated VITEK 2® Compact system (bioMérieux, Marcy-l'Étoile, France) using a GP identification card. Antimicrobial susceptibility tests were performed using the automated VITEK 2® Compact system with the AST-P639 card, and strains showing resistance to nonsusceptibility to at least one agent in three or more antimicrobial categories were classified as multidrug resistant. *Staphylococcus aureus* ATCC29213 was used as a quality control strain.

### 2.2. DNA Extraction

A pure culture colony on blood agar plates was transferred to a 0.5 mL centrifuge tube containing 200 µL proteinase K (200 ng/mL). After one cycle of freezing-thawing (56 °C for 2 h, 95 °C for 10 min), the tubes were centrifuged for 30 s at 24,400 × g to sediment the impurities. The supernatant was stored at −20 °C until use as a template for PCR amplification.

### 2.3. Virulence Gene Detection

A multiplex PCR assay was used to detect virulence genes. Primers were designed based on previous studies [23, 24] and are listed in Table 1. The primers were synthesized by Nanjing Qingke Biotechnology Company (Nanjing, China). The multiplex PCR sample contained 2 µL of DNA template in a 50 µL final reaction mixture of 45 µL of gold (green) and 10 µM upstream and downstream primers. The reaction mixtures were amplified using a thermocycler (Eppendorf, Hamburg, Germany) under the following conditions: initial denaturation at 98 °C for 2 min, followed by 35 cycles of denaturation at 98 °C for 10 s, annealing at 72 °C for 20 s, and extension at 72 °C for 1 min, and a final extension step at 72 °C for 1 min. The PCR products were separated using electrophoresis on a 1.5% agarose gel containing DNA safe stain and visualized using a UV transilluminator. GeneRuler^TM^ 100 bp DNA Ladder was used as a molecular weight marker.

### 2.4. Statistical Analysis

Statistical analyses were performed using the chi-square or Fisher's exact test using SPSS software 16 (SPSS, Inc., Chicago, IL, USA). The results were considered statistically significant when the *P* value was less than 0.05.

## 3. Results and Discussion

### 3.1. Antimicrobial Susceptibility

The antimicrobial resistance profiles for *S. aureus* isolates are listed in Table 2. The resistance rate of 33 *S. aureus* strains to penicillin G was 100% (33/33), to oxacillin was 45.45% (15/33), to erythromycin was 84.85% (28/33), to ciprofloxacin was 72.73% (24/33), and to chloramphenicol was 6.06% (2/33). No strains were resistant to vancomycin, teicoplanin, linezolid, or tigecycline. The frequency of MRSA infection was 45.45% (15/33).

### 3.2. Distribution of Virulence Genes among Methicillin-Susceptible *S. aureus* (MSSA) and MRSA Strains

Seventeen virulence genes were detected among the 33 *S. aureus* strains and are shown in Table 3. The frequency of *hla* and *hlb* was 96.97% (32/33), which was the highest value. The frequency of *lukE* was 51.52% (17/33), while that of *luk-F/S-PV* and *lukM* was 21.21% (7/33), which was the lowest value. The frequency of *splB* was 51.52% (17/33), and the remaining genes encoding protease were all above 81.2% (27/33). The frequencies of *hysA*, *sak*, *lip*, and *nuc* were 30.30% (10/33), 69.70% (23/33), 84.85% (28/33), and 96.97% (32/33), respectively.

The frequency of *hla*, *hlb*, *hlgB*, and *lip* in the MRSA strains was 100% (15/15), and that of *hlgC* was 6.67% (1/15). *Luk-F/S-PV* was not detected in the MRSA strains. The frequency of *hla*, *hlb*, *hld*, *hlgB*, and *lip* among the 18 methicillin-susceptible *S. aureus* (MSSA) strains was 94.44% (17/18), which was the highest value. *luk-F/S-PV* showed the lowest frequency of 5.56% (1/18). The frequencies of *luk-F/S-PV* and *sspB* in the MSSA strains were 5.56% (1/18) and 16.67% (3/18), respectively, which were significantly lower than those in MRSA strains (*P* < 0.05). The frequency of *hlgC* in the MSSA strains was 83.33% (15/18), which was significantly higher than that in the MRSA strains (*P* < 0.05).

### 3.3. Virulence Gene Patterns of 33 *S. aureus* Strains

Twenty-one gene patterns were found among the 33 *S. aureus* strains and are shown in Table 4. Strain nos. 19, 20, and 29 all showed a frequency of 94.00% (16/17) and identical gene patterns. The frequency of strain no. 30 showed the lowest value of 29.00% (5/17). In summary, the six genes encoding hemolysin were found in 29 strains, and the four genes encoding invasive proteases were detected in seven strains.

### 3.4. System Cluster Analysis Results

**Figure 1:**
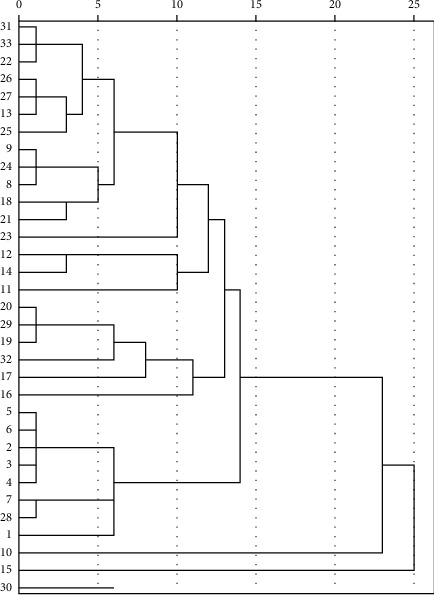
System cluster analysis of 33 *Staphylococcus aureus* virulence genes.

As shown in Figure 1, strain no. 15 and 30 showed high similarity in virulence gene patterns.

### 3.5. Clinical Data of 33 Patients with *S. aureus* Infection

All 33 patients with *S. aureus* infections had different basic diseases; 39.39% (13/33) patients had hypertension, and 33.33% (11/33) had trauma and infections (Table 5). A total of 36.36% (12/33) of patients had fever symptoms and invasive channels. Invasive medical procedures were performed in 100.0% (33/33) of patients. Among them, 24.24% (8/33) and 36.36% (12/33) of patients had undergone percutaneous venous access and placement of drainage tubes, respectively. 9.09% (3/33) and 6.06% (2/33) of patients had been treated to install an indwelling catheter and mechanical ventilation device, respectively. Additionally, 9.09% (3/33) of patients had concomitant infection with Gram-negative bacilli, including *Klebsiella pneumoniae ozaenae* and *Escherichia coli.* We found that 24.24% (8/33) of patients had been treated with more than three antibiotics. A total of 57.57% (19/33) of patients had a good outcome, and 6.06% (2/33) of the patients died (Table 5).

## 4. Discussion

Invasive *S. aureus* infections are considered as important causes of severe sepsis, with higher mortality rates in developing countries than in developed countries [25]. Monitoring results in recent years have shown that the frequency of MRSA infections has been decreasing each year, both domestically and overseas, whereas the proportion of *S. aureus* has not changed significantly [26–30]. The relationship between *S. aureus* antibiotic resistance and virulence genes and human diseases requires in-depth analysis. In this study, we tested the antibiotic resistance and virulence gene distribution of 33 *S. aureus* strains isolated from sterile body fluid samples. The results showed that the frequency of MRSA did not significantly differ from that of the MRSA isolated from various specimens in our hospital from 2011 to 2017. Additionally, the rate of resistance to penicillin G was 100.00% (33/33). The resistance rates of these strains to erythromycin and ciprofloxacin were significantly higher than those of strains from three sample types (blood, pus, and secretions) reported by Zheng [8]. The resistance rates to gentamicin and clindamycin were slightly lower. The resistance rate to chloramphenicol was the lowest. No strains resistant to restricted antibiotics, including vancomycin, teicoplanin, linezolid, and tigecycline, were found, suggesting that use of vanguard-restricted antibacterial drugs such as vancomycin will have effective antibacterial effects based on antimicrobial susceptibility tests.

Our results showed that the frequency of *hla*, *hlb*, *hlgB*, and *nuc* was 96.97% (32/33), which is similar to the results reported by Zhang et al. [31]. All six genes encoding hemolysin were detected in 33 isolates, with the lowest frequency of 87.88% (29/33). Clinical symptoms and image data showed that seven patients had pneumonia. With respect to hospital infectious pneumonia, there is a significant relationship between bacterial colonization specificity, virulence, clinical outcomes, and mortality [32]. In this study, patients who died or had poor prognosis had pulmonary infection and harbored more than 14 virulence genes. The genes encoding leukocidin showed the lowest frequencies among all virulence genes. The frequency of *lukE* was 51.52% (17/33). The frequency of *luk-F/S-PV* was similar to that of the three strains detected in sterile body fluid samples and significantly differed from that of the strains from sputum and wound samples reported in our previous study [20, 21]. The virulence gene carrying rate also showed obvious differences from strains in a group of blood samples abroad [31, 33]. This may be because different specimen types were used. Genes encoding leukocidin were not detected in 13 strains. None of the 13 patients had clinical symptoms or manifestations of pneumonia, except for the patient carrying strain no. 26. It has been reported that 23% of medical workers and 18% of patients are carriers of *Staphylococcus*. Nasopharyngeal *Staphylococcus* carriers are typically the main patients with *Staphylococcus* invasive infections [34, 35]. The frequencies of *hysA*, *sak*, and *lip* were different from those in 19 *S. aureus* strains from blood stream infection in China reported by Zhang et al. [31]. Bacterial virulence genes may be closely related to the evolution of bacteria and host factors [9, 32].

We found that the frequencies of *hla*, *hlb*, and *lip* in the 15 MRSA strains were 100% (33/33) and the frequency of *hlgC* was 6.67% (1/15). Strains carrying *luk-F/S-PV* were not detected. The frequencies of *hla*, *hlb*, *hld*, *hlgB*, and *lip* in the 18 MSSA strains were significantly lower than those in the MRSA strains (*P* < 0.05). The frequency of *hlgC* in the MSSA strains was significantly higher than that in the MRSA strains (*P* < 0.05). There was no significant difference in the remaining virulence genes between the MRSA and MSSA strains. The overall virulence gene carrying rate of MSSA was lower than that of MRSA, which differs from the results of previous studies [36–39]. Whether the cause is related to the specimen type, regional differences, and quantity of specimens should be further examined. We performed systematic cluster analysis on the virulence gene patterns of the 33 *S. aureus* strains, which revealed that the virulence gene patterns of strains no. 15 and 30 were highly similar.

All 33 patients in this study had underlying diseases. All patients had been treated with invasive medical procedures, which is an important cause of infections. The patients were administered more than three types of antibiotics, and most patients infected with MRSA had improved outcomes or were cured. No significant correlation was found between MRSA strain infection and patient death or poor prognosis, which is consistent with previous reports [34, 40]. Studies have reported that *S. aureus*-carrying antibiotic resistance genes may reduce the expression of virulence factors [10, 41]. We did not detect related antibiotic resistance genes. However, there was no significant difference in the frequencies of major virulence genes between the 18 MSSA strains and 15 MRSA strains. Five patients, including one dead and four unhealed, carried more than 14 virulence genes, indicating that the carrying rate of the virulence genes is related to clinical symptoms and outcomes.

## 5. Conclusions

*Staphylococcus aureus* strains are highly resistant to conventional antibacterial drugs. Invasive medical procedures are the main causes of infection. Resistance to antibacterial drugs and the status of virulence genes are highly related to clinical symptoms and outcomes. Further studies are needed to determine the mechanisms of virulence genes and their relation to clinical symptoms and outcomes.

## Figures and Tables

**Table 1:** Virulence factors, genes, primer sequences, and product length.

Virulence factors	Genes	Primer name	Sequence (5′–3′)	Product length (bp)
Hemolysin	*hla*	*hla*-F	ATGAAAACACGTATAGTCAGCTCAGTAACAAC	960
		*hla*-R	TTAATTTGTCATTTCTTCTTTTTCCCAATCGA	
	*hlb*	*hlb*-F	AAATTAGAGCTGAACAAATGAAAGAAATCAG	825
		*hlb*-R	TTTCGCAATTGAATTTGATTGAGGGTCCC	
	*hld*	*hld*-F	ATGGCACAAGATATCATTTCAACAATCAGTG	81
		*hld*-R	TTATTTTTTAGTGAATTTGTTCACTGTGTCG	
	*hlgA*	*hlgA*-F	ATGATTAAAAATAAAATATTAACAGCAACTTTAGCAGTT	860
		*hlgA*-R	TTACTTAGGTGTGATGCTTTTAATTTTTACTTCATGTG	
	*hlgB*	*hlgB*-F	ATGAAAATGAATAAATTAGTCAAATCATCCGTTGC	792
		*hlgB*-R	TTACTGTAATTTTAGATTTTTTAGCGCCATCTTG	
	*hlgC*	*hlgC*-F	GCTTAAAAATAAAATATTAACTACAACTTTATCTGTGAGC	720
		*hlgC*-R	TCAATTCTGTCCTTTCACCTTGATTTCATGAG	
Leukocidin	*lukE*	*lukE*-F	TTGTCAGTAGGACTGATTGCACCTTTAGC	906
		*lukE*-R	TTAATTATGTCCTTTCACTTTAATTTCGTGTGTT	
	*lukM*	*lukM*-F	GTTTAAGAGAAAATTATTAGTTACAACTTTGTCGC	864
		*lukM*-R	GTTGTGCCCCTTTACTTTAATTTCGTG	
	*lukF-PV*	*lukF-PV*-F	ATGAAAAAAATAGTCAAATCATCAGTTGTTACATCA	978
		*lukF-PV*-R	AGCTCATAGGATTTTTTTCCTTAGATTGAG	
	*lukS-PV*	*lukS-PV*-F	GGTCAAAAAAAGACTATTAGCTGCAACATTG	939
		*lukS-PV*-R	TCAATTATGTCCTTTCACTTTAATTTCATGAGTTTTCC	
Invasive proteases	*splB*	*splB*-F	GAACAAAAACGTAGTCATCAAGAGTTTAGCAGC	723
		*splB*-R	CTATGTTTTCTGCAATGAATTTTTTAATTTCTGGTGT	
	*sspA*	*sspA*-F	ATGAAAGGTAAATTTTTAAAAGTTAGTTCTTTATTCGT	783
		*sspA*-R	ATCTTCAATATTTTGTTTTAAGAAGTTGCGTACA	
	*sspB*	*sspB*-F	GTAAATCTAGAGTATTCAATATTATCAGCATCATAATGG	792
		*sspB*-R	AACCTATCATTGAACCATACCAGTTATAATCA	
	*sspC*	*sspC*-F	GTATCAACTACAATTTATAAATTTAGTTTACGACACAACC	330
		*sspC*-R	CTAAGCGCTCATAAACGATTGGGCGC	
Hyaluronidase	*hysA*	*hysA*-F	ATGACATATAGAATGAAGAAATGGCAAAAATTATCCACC	936
		*hysA*-R	TAATTCAAAGCGCACGCCGGATTCATTAGA	
Staphylokinase	*sak*	*sak*-F	ATGCTCAAAAGAGGTTTATTATTTTTAACTGTTTT	492
		*sak*-R	ATTTCTTTTCTATAACAACCTTTGTAATTAAGTTG	
Lipase	*lip*	*lip*-F	GTAGATTATGGTGCAGCACATGCAGCAAAATATGG	921
		*lip*-R	AGCTTTTCAGTTTTCACTAAATCGTCTGCT	
Nuclease	*nuc*	*nuc*-F	AAGAGGTTTTTCTTTTTCGCTACTAGTTGC	537
		*nuc*-R	CTCCAAATATTTAATTTCTGTTGTTTAGCTTT	

**Table 2:** Antimicrobial resistance rate of the 33 *Staphylococcus aureus* strains.

Antibiotics	Resistance rate (*n* = 33) (%)	Intermediary rate (*n* = 33) (%)	Sensitivity rate (*n* = 33) (%)
Penicillin G	33 (100.00)	0 (0.00)	0 (0.00)
Oxacillin	15 (45.45)	0 (0.00)	18 (54.55)
Amoxicillin/clavulanic acid	22 (66.67)	0 (0.00)	11 (33.33)
Gentamicin	13 (39.39)	0 (0.00)	10 (30.30)
Ciprofloxacin	24 (72.73)	0 (0.00)	9 (27.27)
Compound sulfamethoxazole	21 (63.64)	0 (0.00)	12 (36.36)
Clindamycin	15 (45.45)	0 (0.00)	18 (54.55)
Erythrocin	28 (84.85)	0 (0.00)	5 (15.15)
Linezolid	0 (0.00)	0 (0.00)	33 (100.00)
Teicoplanin	0 (0.00)	0 (0.00)	33 (100.00)
Vancomycin	0 (0.00)	0 (0.00)	33 (100.00)
Tigecycline	0 (0.00)	0 (0.00)	33 (100.00)
Chloramphenicol	2 (6.06)	0 (0.00)	31 (93.94)

**Table 3:** Distribution of virulence genes among MSSA and MRSA strains (%).

Virulence genes	Frequency			
	Total number of strains (*n* = 33) (%)	Number of MSSA strains (*n* = 18) (%)	Number of MRSA strains (*n* = 15) (%)	*P* value
*hla*	32 (96.97)	17 (94.44)	15 (100.00)	0.27
*hlb*	32 (96.97)	17 (94.44)	15 (100.00)	0.27
*hld*	31 (93.94)	17 (94.44)	14 (93.33)	0.89
*hlgA*	29 (87.88)	15 (83.33)	14 (93.33)	0.37
*hlgB*	32 (96.97)	17 (94.44)	15 (100.00)	0.27
*hlgC*	29 (87.88)	15 (83.33)	1 (6.67)	≤0.01
*luk-F/S-PV*	7 (21.21)	1 (5.56)	6 (40.00)	0.01
*lukE*	17 (51.52)	8 (44.44)	9 (60.00)	0.37
*lukM*	7 (21.21)	5 (27.28)	2 (13.33)	0.30
*sspA*	30 (90.91)	16 (88.89)	14 (93.33)	0.66
*sspB*	29 (87.88)	3 (16.67)	14 (93.33)	≤0.01
*sspC*	27 (81.82)	16 (88.89)	12 (80.00)	0.48
*splB*	17 (51.52)	8 (44.44)	9 (60.00)	0.37
*hysA*	10 (30.30)	6 (33.33)	4 (26.67)	0.68
*sak*	23 (69.70)	13 (72.22)	11 (73.33)	0.94
*lip*	28 (84.85)	17 (94.44)	15 (100.00)	0.27
*nuc*	32 (96.97)	13 (72.22)	14 (93.33)	0.10

**Table 4:** Virulence gene patterns of 33 *S. aureus* strains.

Serial number	Virulence gene patterns	Strain number	Number of genes (*n* = 17) (%)	Total number of strains
1	*hlb*, *hld*, *sspA*, *sspB*, *sspC*, *sak*	10	6 (0.35)	1
2	*hla*, *hlgB*, *sspB*, *lukM*, *lip*	15	5 (0.29)	1
3	*hla*, *hlb*, *hld*, *hlgB*, *sspA*, *sspB*, *sspC*, *nuc*, *lip*	23	9 (0.53)	1
4	*hla*, *hlb*, *hlgB*, *sspB*, *lip*	30	5 (0.29)	1
5	*hla*, *hlb*, *hld*, *hlgA*, *hlgB*, *hlgC*, *luk-F/S-PV*, *lukE*, *sspA*, *sspB*, *sspC, splB*, *sak*, *nuc*, *lip*	1, 2, 3, 4, 5, 6	15 (0.88)	6
6	*hla*, *hlb*, *hld*, *hlgA*, *hlgB*, *hlgC*, *luk-F/S-PV*, *lukE*, *sspA*, *sspB*, *sspC*, *splB*, *nuc*, *lip*	7	14 (0.82)	1
7	*hla*, *hlb*, *hld*, *hlgA*, *hlgB*, *hlgC*, *sspA*, *sspB*, *sspC*, *sak*, *nuc*, *lip*, *hysA*	8, 9	12 (0.71)	2
8	*hla*, *hlb*, *hld*, *hlgA*, *hlgB*, *hlgC*, *lukM*, *sspC*, *splB*, *sak*, *lip*	11	11 (0.65)	1
9	*hla*, *hlb*, *hld*, *hlgA*, *hlgB*, *hlgC*, *lukM*, *sspA*, *sspB*, *sspC*, *splB*, *lip*	12	12 (0.71)	1
10	*hla*, *hlb*, *hld*, *hlgA*, *hlgB*, *hlgC*, *sspA*, *sspB*, *sspC*, *sak*, *nuc*, *lip*	13, 26, 27	12 (0.71)	3
11	*hla*, *hlb*, *hld*, *hlgA*, *hlgB*, *hlgC*, *sspA*, *sspB*, *sspC*, *splB*, *lip*	14	11 (0.65)	1
12	*hla*, *hlb*, *hld*, *hlgA*, *hlgB*, *hlgC*, *lukE*, *sspA*, *sspC*, *splB*, *nuc*, *lip*, *hysA*	16	13 (0.76)	1
13	*hla*, *hlb*, *hld*, *hlgA*, *hlgB*, *hlgC*, *lukM*, *sspA*, *sspB*, *sspC*, *splB*, *sak*, *nuc*, *lip*	17	14 (0.82)	1
14	*hla*, *hlb*, *hld*, *hlgA*, *hlgB*, *hlgC*, *sspA*, *sspB*, *sspC*, *nuc*, *lip*, *hysA*	18	12 (0.71)	1
15	*hla*, *hlb*, *hld*, *hlgA*, *hlgB*, *hlgC*, *lukE*, *lukM*, *sspA*, *sspB*, *sspC*, *splB*, *sak*, *nuc*, *lip*, *hysA*	19, 20, 29	16 (0.94)	3
16	*hla*, *hlb*, *hld*, *hlgA*, *hlgB*, *hlgC*, *sspA*, *sspB*, *sspC*, *nuc*, *lip*	21	11 (0.65)	1
17	*hla*, *hlb*, *hld*, *hlgA*, *hlgB*, *hlgC*, *sspA*, *sspB*, *sak*, *nuc*, *lip*	22, 31, 33	10 (0.59)	3
18	*hla*, *hlb*, *hld*, *hlgA*, *hlgB*, *hlgC*, *sspA*, *sspB*, *sspC*, *sak*, *nuc*, *lip*, *hysA*	24	14 (0.82)	1
19	*hla*, *hlb*, *hld*, *hlgA*, *hlgB*, *hlgC*, *sspA*, *sspC*, *sak*, *nuc*, *lip*	25	11 (0.65)	1
20	*hla*, *hlb*, *hld*, *hlgA*, *hlgB*, *hlgC*, *luk-F/S-PV*, *lukE*, *sspA*, *sspB*, *sspC*, *splB*, *nuc*, *lip*	28	14 (0.82)	1
21	*hla*, *hlb*, *hld*, *hlgA*, *hlgB*, *hlgC*, *lukE*, *lukM*, *sspA*, *splB*, *sak*, *nuc*, *lip*, *hysA*	32	14 (0.82)	1

**Table 5:** Clinical data of 33 patients with *S. aureus* infection.

Clinical information		Number of patients (*n* = 33) (%)
Basic disease (7)	Diabetes	6 (18.18)
	Hypertension	7 (21.21)
	Cardiovascular disease	1 (3.03)
	Uremia	1 (3.03)
	Trauma	5 (15.15)
	Respiratory infection	5 (15.15)
	Urinary tract infection	1 (3.03)

Sign (2)	Fever	23 (69.70)
	Chest film/CT with positive sign	8 (24.24)

Invasive medical procedures (4)	Percutaneous venous access	8 (24.24)
	Drainage tube	12 (36.36)
	Indwelling catheter	3 (9.09)
	Mechanical ventilation	2 (6.06)
Concomitant infection (1)	Gram-negative bacilli infection	3 (9.09)

Outcome (5)	Cure	4 (12.12)
	Improved	15 (45.45)
	Automatic discharge	5 (15.15)
	Death	2 (6.06)
	Other	2 (6.06)

Treated with more than three antibiotics		8 (24.24)

## Data Availability

The data used to support the findings of this study are included within the article.
